# Where Reflectance Confocal Microscopy Provides the Greatest Benefit for Diagnosing Skin Cancers: The Experience of the National Cancer Institute of Naples

**DOI:** 10.3390/cancers17111745

**Published:** 2025-05-22

**Authors:** Marco Palla, Gerardo Ferrara, Corrado Caracò, Luigi Scarpato, Anna Maria Anniciello, Paolo Meinardi, Alfonso Amore, Rossella Di Trolio, Giuseppina Marano, Benedetta Alfano, Manuel Tuccillo, Domenico Mallardo, Giovanni Pellacani, Paolo Antonio Ascierto

**Affiliations:** 1 Department of Melanoma, Cancer Immunotherapy and Development Therapeutics, Istituto Nazionale Tumori IRCCS “Fondazione G. Pascale”, 80131 Naples, Italy; l.scarpato@istitutotumori.na.it (L.S.); r.ditrolio@istitutotumori.na.it (R.D.T.); giuseppina.marano@istitutotumori.na.it (G.M.); benedetta.alfano@istitutotumori.na.it (B.A.); d.mallardo@istitutotumori.na.it (D.M.); p.ascierto@istitutotumori.na.it (P.A.A.); 2 Department of Pathology and Cytopathology, Istituto Nazionale Tumori IRCCS “Fondazione G. Pascale”, 80131 Naples, Italy; gerardo.ferrara@istitutotumori.na.it (G.F.); a.anniciello@istitutotumori.na.it (A.M.A.); 3 Division of Surgery of Melanoma and Skin Cancer, Istituto Nazionale Tumori IRCCS “Fondazione G. Pascale”, 80131 Naples, Italy; corrado.caraco@istitutotumori.na.it (C.C.); p.meinardi@istitutotumori.na.it (P.M.); a.amore@istitutotumori.na.it (A.A.); 4 Management Control and Information Services, Istituto Nazionale Tumori IRCCS “Fondazione G. Pascale”, 80131 Naples, Italy; manuel.tuccillo@istitutotumori.na.it; 5 Dermatology Clinic, Department of Clinical Internal, Anesthesiologic and Cardiovascular Sciences, Sapienza University of Rome, 00185 Rome, Italy; pellacani.giovanni@uniroma1.it

**Keywords:** melanocytic lesions, melanoma, epiluminescence microscopy, reflectance confocal microscopy

## Abstract

**Background**: Although complete excision of suspicious melanocytic lesions is mandatory, it carries the risk of unnecessary scarring on one hand and inadequate treatment of misdiagnosed lesions on the other. **Objectives**: We evaluated the sensitivity, specificity, and predictive value of reflectance confocal microscopy (RCM) in diagnosing pigmented lesions with clinically ambiguous features―the so-called “gray zone” ―and compared its performance with the more established technique of epiluminescence microscopy (ELM). **Results**: Between 2019 and 2020, a total of 2282 melanocytic lesions were assessed using both ELM and RCM. Histopathological diagnosis aligned with the ELM risk classification in 91.6% of melanocytic lesions, specifically in 92.0% of very-high-risk lesions, 88.5% of high-risk lesions, 66.3% of medium-risk lesions, 96.3% of low-risk lesions, and 98.0% of very low-risk lesions. Similarly, histopathological diagnosis of these lesions corresponded with the RCM risk assessment in 91.2% of cases, including 90.9% of very-high-risk lesions, 84.4% of high-risk lesions, 93.1% of medium-risk lesions, 90.5% of low-risk lesions, and 96.2% of very low-risk lesions. **Conclusions**: Although ELM is a valuable tool for increasing the efficacy of clinical diagnosis, its reliability decreases for a group of lesions that appear suspicious during clinical skin examination. RCM, as a newer technique, appears to improve malignancy detection in suspicious melanocytic lesions without requiring excision; its sensitivity and specificity remain high even in lesions classified by ELM as posing a medium risk of malignancy.

## Simple Summary

In case of suspicious skin spots, it is important to remove them if there is a chance they could be cancerous. However, unnecessary removal can leave scars, while missing a cancer diagnosis can be dangerous. This study looked at a new imaging method called reflectance confocal microscopy, which lets doctors examine skin at a cellular level without surgery. The goal was to see how well this method detects skin cancer in lesions that are difficult to classify with standard examination. We compared it with a more established tool, called dermoscopy, across over 2000 skin lesions. The results showed that both tools are helpful, but reflectance confocal microscopy was especially accurate for skin spots that are not clearly benign or malignant. This research suggests that this non-invasive technique could help doctors better decide which lesions need to be removed, reducing unnecessary surgeries and improving early cancer detection.

## 1. Introduction

Despite the availability of new treatments, advanced melanoma continues to have a severe prognosis. Early diagnosis, followed by surgical excision with appropriate margins, remains the most effective strategy to reduce mortality [1,2]. Although the complete excision of suspicious lesions is mandatory, it carries the risk of unnecessary scarring on one hand and inadequate treatment of misdiagnosed lesions on the other. As a result, non-invasive imaging techniques have been proposed to support the clinical examination and improve the efficiency of presurgical diagnosis.

Dermoscopy, also known as epiluminescence microscopy (ELM), has become an established technique over the past decades, while reflectance confocal microscopy (RCM) is the most recent method to achieve a high level of evidence (1b) and a grade B recommendation for melanoma diagnosis in the European guidelines [3,4,5]. Current available diagnostic criteria for clinical examination and imaging techniques remain unable to perfectly distinguish all pigmented lesions as either benign or malignant, which represent a significant proportion of cases encountered in dermatologic practice. A key challenge is to reduce this so-called “gray zone” of melanocytic lesions by optimizing resource utilization [4,6].

RCM is an optical imaging technique that enables the vivo visualization of cutaneous lesions down to the depth of the papillary dermis (approximately at a thickness of 300 mm), with cellular level resolution of 0.5–1.0 μm laterally and 4.0–5.0 μm axially, without requiring a biopsy for histological assessment [7,8,9]. It is used to examine melanocytic lesions, as melanin and melanosomes provide an efficient source of contrast, facilitating the detection of melanocytic cells [10,11]. Despite the operator dependence and limited penetration depth, the implementation of RCM as a complementary tool to dermoscopy is particularly relevant in these scenarios, where its high-resolution, non-invasive imaging capabilities may reduce both the number of unnecessary excisions and the risk of missing melanomas [12,13,14]. However, real-world data evaluating the added diagnostic value of RCM in lesions categorized as medium risk—those that most frequently challenge clinical decision-making—are still limited.

To optimize the use of imaging techniques in the early diagnosis of melanoma, we evaluated the sensitivity, specificity, and predictive value of RCM in diagnosing pigmented lesions with clinically ambiguous features, the so-called “gray zone”, and compared it with the more established technique of ELM.

## 2. Patients and Methods

### 2.1. Workflow

**Figure 1:**
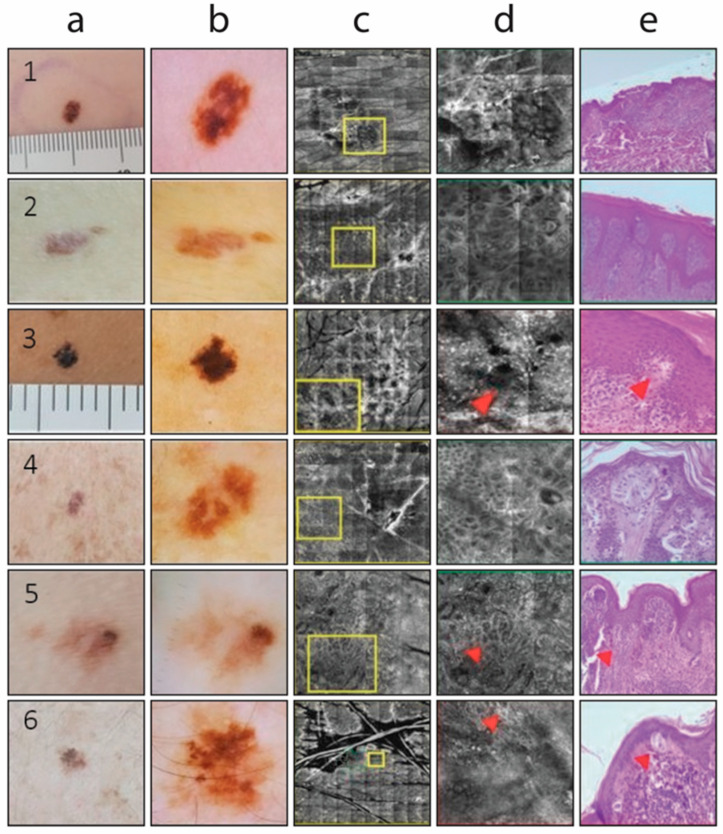
Some examples of clinical (columns a,b), RCM (columns c,d), and histological (column e) images of type-3 pigmented lesions. Columns a,b show clinical and dermoscopic (ELM) images, respectively. Dermoscopic images highlight key features used for risk stratification, such as pigment network irregularities, asymmetry in color or structure, and the presence of melanoma-associated patterns including pseudopods, radial streaming, and blue-gray veil. Column c presents RCM images at the dermoepidermal junction, where architectural disarray, presence of atypical bright cells, and irregular nests are assessed. Column d shows magnified areas of interest; red arrows correspond to the same locations of the cross-sectional images in column e. The red rectangle indicates areas where melanoma-specific confocal features are present, including disarray of the dermoepidermal junction and atypical dendritic or round cells. Column e shows corresponding histopathological images used as reference standards. Rows 1, 2, 4, and 5: dysplastic nevus; rows 3 and 6: in situ melanoma on nevus.

In 2019 and 2020, the protocol of the Dermatology and Oncology Units of the National Cancer Institute of Naples, Italy, included a clinical visit with total-body skin examination (TBSE) to evaluate cutaneous pigmented lesions, followed by ELM assessment of clinically suspicious lesions. Non-melanocytic lesions were excluded from further evaluation. Based on ELM evaluation, lesions recommended for surgical excision because of suspected malignancy were also assessed using RCM (Figure 1).

Lesion assessment was based on the evaluation of standardized and reproducible atypical RCM features, as identified by experienced and skilled operators according to international consensus criteria [15]. During clinical evaluation, cutaneous melanocytic lesions were classified according to the macroscopic ABCDE criteria [16]. ELM assessments were performed by four expert dermatologists (each with at least 5 years of experience) using the VivaScope^®^ 1500 RCM system (Caliber Imaging and Diagnostics, Rochester, NY, USA) [5]. Lesions were classified according to the ELM-based risk criteria for cutaneous pigmented lesions, previously published by our group [17].

Patients recommended for surgery of a melanocytic lesion were invited to participate in our study after providing written informed consent. This study was reviewed and approved by the Ethics Committee at the National Cancer Institute of Naples (Italy) (protocol no. 33/17 approved on 10 January 2018).

The pathologic diagnosis served as the reference standard for evaluating the sensitivity and specificity of RCM and ELM.

### 2.2. Lesion Classification at ELM

Table 1 illustrates the ELM-based risk classification applied in this study to categorize melanocytic lesions according to their dermoscopic appearance and associated risk of malignancy. Type-1 lesions were considered at very high risk, displaying classic dermoscopic hallmarks of melanoma. Type-2 lesions, classified as high risk, exhibited more subtle yet concerning features that could overlap with those seen in atypical nevi, including asymmetries in color or structure and irregular pigment networks. Type-3 lesions, representing the medium-risk category, showed mild architectural irregularities of the pigment network that could correspond either to benign dysplastic nevi or early melanomas. Lesions classified as type 4 (low risk) and type 5 (very low risk) exhibited dermoscopic features typically associated with benignity, such as regular pigment networks or globular patterns, and were considered to have a low or minimal probability of malignancy, respectively. ELM diagnoses were compared with histopathological diagnosis, which served as the reference parameter. In cases of diagnostic uncertainty, additional immunohistochemical stains (SOX10, MART1, S100) were used to reach a definitive diagnosis.

### 2.3. Reflectance Confocal Microscopy (RCM) Assessment

Following ELM evaluation, melanocytic lesions were examined using RCM. All lesions were described using standardized terminology based on the Consensus on Confocal Reflectance Imaging [18,19] and evaluated with the Modena algorithm—an RCM score system for diagnosing malignant melanoma [20].

Briefly, a lesion was classified as “probable melanoma” if it exhibited dermoepidermal junction (DEJ) disarray in >10% of cells, along with the presence of atypical cells. Melanoma was suspected if the lesions had DEJ disarray in >10% of cells with atypical cells present. Lesions without atypical cells and DEJ disarray were classified as “melanoma not suspected”.

### 2.4. Statistical Methods

Statistical analyses for ELM and RCM sensitivity, specificity, and predictive values were performed by computer-assisted statistical analysis using SPSS software for Windows, version 8.0 (SPSS Inc., Chicago, IL, USA). Histopathology was used as the reference standard for diagnostic accuracy. A lesion was classified as true positive (TP) or true negative (TN) if RCM or ELM results were consistent with histology (in other words, concordance was obtained using these two diagnostic approaches). Conversely, a lesion was classified as false positive (FP) or false negative (FN) if histology did not confirm the RCM/ELM classification (in other words, RCM/ELM was positive, but histology was negative, or RCM/ELM was negative, but histology was positive). Sensitivity was calculated as TP/TP +FN and specificity as TN/TN + FP. The positive predictive value was calculated as TP/TP + FP, and the negative predictive value as TN/TN + FN. Receiver operating characteristic (ROC) curves were used to evaluate the diagnostic performance of the tested model. These curves plot the true positive rate (sensitivity) against the false positive rate (1-specificity) across various threshold settings. The area under the curve (AUC) was calculated as a measure of overall discriminatory accuracy, with values closer to 1 indicating better performance. Raw data generated in this study have been deposited in Zenodo: https://doi.org/10.5281/zenodo.14945999.

## 3. Results

During 2019–2020, a total of 3451 TBSEs were performed at the National Cancer Institute of Naples for the early diagnosis of malignant melanoma. Of the evaluated lesions, 1169 were non-melanocytic, and 2282 were melanocytic. Among the melanocytic lesions, 657 were diagnosed as melanomas. Breslow thickness measurements for melanomas are reported in Table 2.

According to ELM criteria (Table 3), the 2282 melanocytic lesions were classified as very high risk (*n* = 364; 15.9%), high risk (*n* = 468; 20.5%), medium risk (*n* = 235; 10.2%), low risk (*n* = 463; 20.2%), and very low risk (*n* = 752; 32.9%).

The histopathological diagnosis was consistent with ELM classification in 91.6% of cases, specifically in 92.0% of very-high-risk lesions, 88.5% of high-risk lesions, 66.3% of medium-risk lesions, 96.3% of low-risk lesions, and 98.0% of very-low-risk lesions (Table 3).

Before surgery, all 2282 melanocytic lesions were further evaluated using RCM. The histopathological diagnosis was in agreement with RCM classification in 91.2% of cases, specifically in 90.9% of very-high-risk lesions, 84.4% of high-risk lesions, 93.1% of medium-risk lesions, 90.5% of low-risk lesions, and 96.2% of very-low-risk lesions (Table 3).

**Figure 2:**
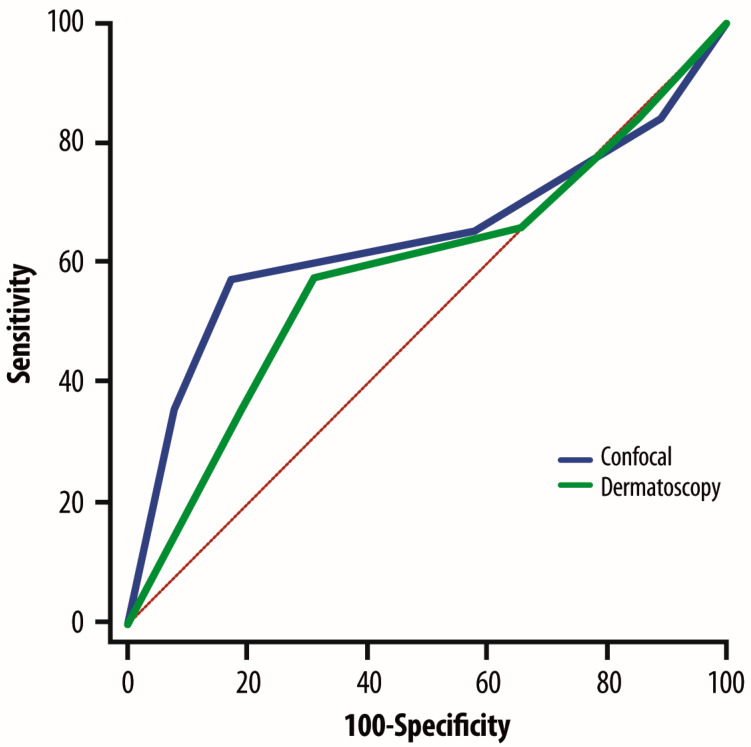
ROC curves showing specificity and sensitivity of ELM and RCM. Red line: Random reference line.

The specificity and sensitivity of ELM and RCM were compared using ROC curves. RCM approach was confirmed to be better than ELM (AUC = 0.64 vs. 0.59; 95% CI: 0.028–0.081; *p* = 0.0001) (Figure 2).

In particular, ELM demonstrated poorer sensitivity and specificity in medium-risk lesions than in the other risk categories (Table 4). Notably, in this subgroup, specificity was lower than sensitivity when compared with histopathology (55.7% vs. 73.5%; K = 0.296; *p* < 0.0001).

In contrast, RCM assessment of lesions classified at medium risk by ELM showed a diagnostic concordance with histopathology in 93.1% of cases, demonstrating high sensitivity and specificity. Specifically, RCM sensitivity and specificity in medium-risk lesions were superior to those of ELM (92.4 vs. 73.5 and 94.0 vs. 55.7, respectively).

## 4. Discussion

This study evaluated the sensitivity and specificity of two imaging techniques for the early diagnosis of melanocytic lesions, comparing the clinical assessment with the histopathological diagnosis. Although dermoscopy shows slightly higher overall concordance in absolute numbers, the ROC curve analysis—which evaluates sensitivity and specificity across a continuous scale—demonstrates superior performance of RCM in the diagnostically challenging mid-risk category. Indeed, the ROC curve showed that patients evaluated with RCM had greater diagnostic power in terms of specificity and sensitivity than those evaluated with ELM alone. In particular, the proportion of FP-positive and FN cases in ELM evaluation was higher for lesions classified as medium risk than those classified in other risk categories. This observation indicates that although ELM is a valuable tool for increasing the effectiveness of clinical diagnosis, it is less reliable in a group of lesions that appear suspicious during clinical skin examination. This means that ELM is less useful precisely in cases where objective observation mainly needs support. The newer technique of RCM appears to provide a more significant improvement in detecting malignancy in equivocal melanocytic lesions as its sensitivity and specificity remain high even in lesions that ELM classifies as posing a medium risk of malignancy. Thus, RCM is confirmed as a reliable technique for the clinical evaluation of pigmented lesions, helping to reduce unnecessary surgeries or missed diagnoses of malignancy.

Several studies have confirmed the overall sensitivity and specificity of RCM for melanoma diagnosis. A meta-analysis of eight studies found that RCM had higher diagnostic accuracy for melanoma than ELM [21]. The pooled sensitivity of ELM for melanoma detection was 88.4% (95% CI: 0.84–0.92), whereas the pooled sensitivity of RCM was 93.5% (95% CI: 0.90–0.96). The pooled specificity of ELM for melanoma detection was 49.1% (95% CI: 0.84–0.92), while that of RCM was 78.8% (95% CI: 0.75–0.82). Similarly, a systematic review by Lan et al. [21], which included seven studies, found that RCM was superior to ELM for melanoma detection even in challenging cases, such as amelanotic and hypomelanotic lesions, with 67% (95% CI: 0.51–0.81) sensitivity and 89% (95% CI: 0.86–0.92) specificity.

The present study specifically evaluates the contribution that RCM may provide in the diagnosis not only of all pigmented lesions but, more importantly, those that remain uncertain based on clinical examination. Our results suggest that RCM is superior to ELM in this subset of lesions. These data align with the results of a recent randomized clinical trial [22], which showed that the adjunctive use of RCM for suspicious lesions halved the number of unnecessary excisions. In a similar approach, the authors defined their inclusion criteria as lesions with a primary diagnostic suspicion of “malignant” or “undefined/uncertain/just guessing”, with a confidence level of “probable” or “possible” [23]. These criteria align with what could be considered a clinical classification of “medium risk” for melanoma. Nevertheless, the efficiency of ELM is very high, and the two techniques should be used as additional tools, according to the findings of the review by Marushchak et al. [24].

The features of ELM and RCM are mutually complementary: RCM can detect subtle clues of in situ melanoma in superficial layers that may not be apparent on ELM. Additionally, RCM uses the refractive properties of subsurface structures and may detect amelanotic lesions, which ELM may not visualize [5,24]. Future research should focus on prospective, multicenter studies to validate these findings across diverse clinical settings and operator expertise levels. Additionally, the integration of RCM with emerging technologies such as artificial intelligence-based image analysis holds promise for further enhancing diagnostic accuracy, reducing operator dependency, and standardizing assessments [25,26,27]. Moreover, economic evaluations are warranted to determine the cost-effectiveness of implementing RCM in routine practice, particularly in the management of lesions within the diagnostic gray zone. Finally, educational initiatives aimed at increasing clinician proficiency in RCM interpretation could further optimize its use and broaden its applicability, ultimately improving patient outcomes by ensuring more accurate and less invasive melanoma diagnosis.

## 5. Conclusions

In conclusion, RCM enhances the clinical diagnosis of melanocytic lesions by improving specificity and sensitivity. It provides additional diagnostic accuracy to ELM, which may not be sufficient to rule out false-positive and false-negative cases in a subgroup classified as medium risk.

## Figures and Tables

**Table 1:** ELM-based risk classification for cutaneous pigmented lesions.

Melanocytic Lesions	ELM Features
Type 1 (very high risk)	Lesion with a pigment network and distinct ELM features strongly associated with melanoma, such as pseudopods, radial streaming, a blue-gray veil, or atypical vessels.
Type 2 (high risk)	Lesion with a pigment network and subtle emerging ELM features that may indicate melanoma but are also commonly found in atypical nevi.
Type 3 (medium risk)	Lesion with a pigment network showing mild structural irregularities, which can be observed in atypical nevi and melanocytic hyperplasia.
Type 4 (low risk)	Lesion with a uniformly benign-appearing pigment network.
Type 5 (very low risk)	Lesion with a benign-appearing pigment network, often exhibiting a globular pattern or another characteristic benign ELM structure.

**Table 2:** Melanoma cases according to Breslow thickness.

Breslow Thickness Categories	Cases (*n* = 657), n	Median Breslow Thickness (mm)	Range (mm)
Diffuse intraepidermal atypical cells, melanoma in situ, MELTUMP	93	No	No
<0.8 mm	257	0.5	0.3–0.8
<0.8 mm with ulceration or 0.8–1.0 mm	189	0.9	0.8–1
>1.1–2.0 mm	153	1.65	1.3–2.0
>2.1–4.0 mm	39	2.45	2.1–2
>4.0 mm	19	4.65	>4

MELTUMP: Melanocytic tumors of uncertain malignant potential.

**Table 3:** Agreement of diagnosis of either ELM or RCM with histological diagnosis.

ELM Levels of Risk	N (no. of MMs, %)	ELM Agrees with Histology, n (%)	RCM Agrees with Histology, n (%)
Very high	364 (15.9)	335 (92.0)	330 (90.9)
High	468 (20.5)	414 (88.5)	394 (84.4)
Medium	235 (10.3)	156 (66.3)	219 (93.1)
Low	463 (20.4)	450 (96.3)	418 (90.5)
Very low	752 (32.9)	737 (98.0)	722 (96.2)
Total	2282	2092	2083

MMs: malignant melanomas.

**Table 4:** Sensitivity and specificity of either ELM or RCM in different ELM levels of risk.

ELM Levels of Risk	N	ELM Sensitivity	ELM Specificity	RCM Sensitivity	RCM Specificity
Very high	364	98.4	78.2	95.6	68.2
High	468	93.8	70.6	88.6	67.6
Medium	235	73.5	55.7	92.4	94.0
Low	463	98.5	85.1	92.9	85.3
Very low	752	98.3	83.3	96.6	81.8

## Data Availability

All data supporting the findings of this study are included in the article. Further inquiries can be directed to the corresponding author upon reasonable request. The data presented in this study are openly available in Zenodo at https://doi.org/10.5281/zenodo.14945999.
